# Coronary Embolism and Myocardial Infarction in a Transgender Male Undergoing Hormone Therapy: A Case Report and Review of the Literature

**DOI:** 10.1155/2020/4829169

**Published:** 2020-03-23

**Authors:** Sandesh Dinesh, Marcus Franz, Friedhelm Küthe

**Affiliations:** ^1^Ilmkreis-Kliniken, Clinic for Internal Medicine I, Bärwinkelstrasse 33, 99310 Arnstadt, Germany; ^2^Department of Internal Medicine I, University Hospital Jena, Am Klinikum 1, 07747 Jena, Germany

## Abstract

*Introduction*. Due to the complex interaction between the underlying disease, psychosocial factors, and the high-dose hormonal therapy, transgender patients pose a therapeutic and diagnostic challenge, especially during emergencies. This case presents one such clinical dilemma using the example of a case of myocardial infarction.

**Case:**

A 35-year-old transgender male presented to our clinic with an acute inferior wall myocardial infarction. For the past 6 years, he was receiving high-dose testosterone therapy for the maintenance of hormone levels after female-to-male gender conversion. The emergency coronary angiography revealed a distal right coronary artery occlusion. Recanalization of the vessel was achieved by catheter-driven direct thrombectomy and subsequent intracardiac lysis. The appearance of the remaining coronary arteries bore no angiographic evidence of advanced coronary artery disease. We suspected a thromboembolic origin as the primary cause of the myocardial infarction. The presentation also fulfilled the proposed National Cerebral and Cardiovascular Center criteria for the clinical diagnosis of coronary embolism. In the diagnostic work-up, the most common causes of coronary embolism like atrial fibrillation, cardiomyopathies, endocarditis, and intracardiac tumors could be ruled out. The screening for hereditary thrombophilia was also negative. Likewise, the presence of a haemodynamically relevant right to left shunt could be excluded. In the end, the high-dose testosterone therapy seemed to be the most likely cause.

**Conclusion:**

Following major thromboembolic cardiovascular events, we believe that transgender males treated with high-dose testosterone therapy should receive oral anticoagulation, preferably with a DOAC, especially keeping in mind that the discontinuation of the hormone therapy is not always possible due to the various underlying psychosocial factors.

## 1. Case Presentation

**Figure 1:**
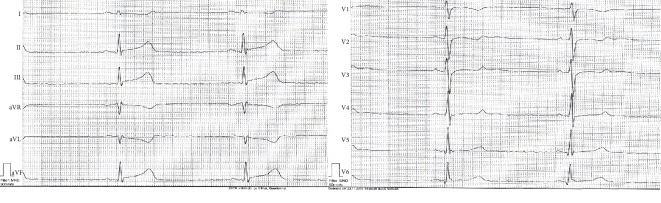
Electrocardiogram of the patient showing sinus rhythm with ST-Segment elevation in the inferior leads.

A transsexual 35-year-old Caucasian male without known relevant medical history of cardiac disease presented to the emergency room with a sudden onset of typical anginal chest pain. Physical examination revealed a hemodynamically stable patient with resting pulse of 70/min and blood pressure of 110/70 mmHg, with no signs of acute cardiopulmonary decompensation. Cardiac examination was uneventful with normal heart sounds without pathological murmurs. Respiratory, abdomen, and neurological examinations remained normal. Active smoking accumulating to 20 pack years and a history of daily alcohol consumption were significant risk factors. Drug history revealed a hormonal therapy with intramuscular testosterone undecanoate for gender conversion over the past 6 years, with an initial dosage of 1000 mg per month over a period of 1 year from 2011 to 2012 and later at a dose of 1000 mg every 3 months to date. An electrocardiogram showed sinus rhythm with ST-Segment elevation in the inferior leads (Figure 1).

**Figure 2:**
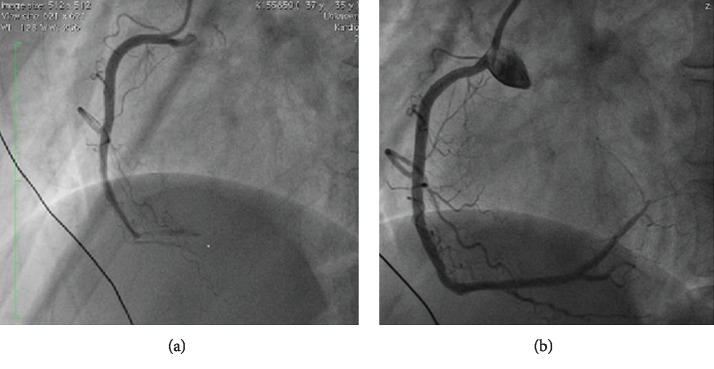
Coronary angiography of the patient revealing normal coronary arteries with an occlusion of the distal right coronary artery (RCA) before (a) and after successful recanalization (b).

An emergency coronary angiography revealed normal coronary arteries with an occlusion of the distal right coronary artery (Figure 2(a)). The appearance of the remaining coronary arteries was smooth, not indicative of an advanced atherosclerotic coronary artery disease. We suspected a thromboembolic origin as the primary cause of the myocardial infarction by the appearance of the coronaries. Recanalization of the vessel was achieved by catheter-driven direct thrombectomy using an aspiration catheter. This was followed by intracardiac lysis with 20 mg tissue plasminogen activator, Alteplase, and subsequently the intravenous glycoprotein IIb/IIIa inhibitor Tirofiban. A short episode of reperfusion-related ventricular fibrillation and subsequent third-degree atrioventricular block (av-block) were overcome by defibrillation and intravenous administration of 2 mg Atropine, respectively. Finally, a successful recanalization was achieved with TIMI-3 flow (Figure 2(b)) with immediate resolution of the patient's symptoms. A stent implantation was not necessary. Cardiac biomarkers were subsequently elevated with Troponin I up to a maximum of 46.63 ng/ml (Reference range: 0.00–0.06 ng/ml). Echocardiography revealed a good systolic left ventricular function with postinfarct inferior wall hypokinesia. The patient remained haemodynamically stable without complications during the hospital stay.

Subsequently, investigations were conducted with the aim to identify an underlying cause for the suspected embolic myocardial infarction. The transthoracic echocardiography did not reveal intracardiac thrombus or valve vegetations as evidence for infective endocarditis. A deep venous thrombosis could also not be detected by ultrasonography, and the D-Dimer levels were negative.

The initially performed transoesophageal echocardiography under sedation (Midazolam) raised the suspicion of a possible small persisting foramen ovale (PFO). We performed a second contrast-enhanced TOE without sedation. No crossover of microbubbles over to the arterial circulation were observed even under Valsalva manoeuvre. Thus, a haemodynamically relevant right to left shunt could be ruled out.

No evidence of paroxysmal atrial fibrillation was reported in a 72 h Holter-ECG Monitoring. Cardiac embolic sources were thus deemed unlikely.

Other manifestations of atherosclerotic vessel diseases could not be seen in the colour-coded Doppler Ultrasound scans of the extracranial arteries or pelvic/lower limb arteries.

Laboratory tests for Antiphospholipid Syndrome (APS), Factor-V Leiden, Protein C and Protein S deficiency, Antithrombin III deficiency, and Hyperhomocysteinemia as possible causes for a hypercoagulable state were also negative.

In conclusion, the work-up for the definitive cause of the infarction remained inconclusive. The drug history of the patient with the use of high-dose hormonal therapy with testosterone for gender conversion was one of the significant factors in the patient's history.

This background of high-dose testosterone therapy for the purpose of gender conversion and its procoagulant associations led us believe this to be the factor leading to the coronary embolism.

A follow-up of the patient was performed at 3 months and 1 year. The echocardiography and ECG showed no signs of long-term detrimental effects following the myocardial infarction with resolution of the initial wall motion disturbances.

## 2. Discussion and Review of the Literature

Transgender patients undergoing sex change therapy pose a unique challenge with a complex interaction of underlying disease, psychosocial factors, and the additional high-dose hormone therapy. With the number of cases of gender conversion gradually increasing [1], the altered hormone milieu due to the hormone therapy in this patient group and its possible side effects like thromboembolism must be given special consideration.

In this case, we present one such diagnostic and therapeutic dilemma. At initial presentation, our patient met the diagnostic criteria for an acute inferior wall ST-elevation myocardial infarction (STEMI). Except for a significant smoking history accumulating to 20 pack years, there were no other known risk factors in the patient's past history. Due to the young age of the patient and the medical history, we thought of an alternative explanation rather than an atherosclerotic disease as the underlying cause for the acute myocardial infarction. The coronary angiogram supported our uncertainty with evidence of normal coronary arteries and with no evidence of underlying atherosclerosis. Other invasive coronary imaging modalities like optical coherence tomography or intravenous ultrasound were not performed in this patient, although these modalities would have helped in definitively ruling out significant atherosclerotic plaques. Other signs of systemic atherosclerotic vessel disease could also be ruled out in the subsequent colour-coded Doppler ultrasound scans of the extracranial arteries and pelvic/lower limb arteries.

The myocardial infarction was hypothesized to be of embolic origin, due to a coronary embolism. The subsequent successful treatment with catheter-directed thrombectomy and intracardiac lysis with Tirofiban, along with no evidence of plaque rupture, underlined the case for an embolic origin of the episode. The clinical case satisfying one major criteria with angiographic evidence of coronary embolism without atherosclerotic component and two minor criteria with <25% stenosis on angiography, except for the culprit lesion and the presence of an embolic risk in the form of hypercoagulable state in our case, of the proposed National Cerebral and Cardiovascular Center criteria for the clinical diagnosis of coronary embolism by Shibata and coworkers [2] also backed our clinical hypothesis.

Our further work-up was aimed at finding a causative link for the coronary embolism. Literature search showed the most common factors leading to coronary emboli in the order of descending significance to be (a) atrial fibrillation, (b) dilated cardiomyopathy, (c) endocarditis, and (d) intracardiac tumors [3]. Other systemic prothrombotic diseases like active malignancies or systemic autoimmune diseases are also found to be responsible for coronary embolism. Among the various causes, atrial fibrillation remained the most common cause in adults, including a subset of the patient population having new-onset AF, not on anticoagulation.

Given the known history of smoking and alcohol consumption of our patient [4], he possessed many of the risk factors associated with atrial fibrillation. Despite this, there was no documented evidence of atrial fibrillation. Nonetheless, repeated events of asymptomatic self-limiting paroxysmal AF occurring cannot completely be ruled out. An implantable cardiac loop recorder increases the chances of documentation of such episodes. Thus, at least in patients with cerebral embolism, the guideline for the management of atrial fibrillation published by the European society of cardiology in 2016 recommends the implantation as IIa indication with a level of evidence B [5]. For coronary embolism, a corresponding recommendation does not exist to our best knowledge. Therefore, after internal discussion, we decided against the implantation of a loop recorder. The other potential causes for coronary embolism mentioned above could be ruled out through further work-up as described. Among others, since cases of venous thromboembolism presenting as coronary embolism due to paradoxical embolization over a PFO have been well documented in the literature [6], special emphasis was given on the exclusion of this constellation in our patient by ruling out lower limb deep venous thrombosis and a PFO or a atrial septal defect.

Finally, the search for the cause for the coronary embolism failed to clearly identify the underlying aetiology of the event. An important factor at this point seemed to be the high-dose testosterone therapy for gender conversion causing the activation of the thrombotic cascade in the arterial circulation, leading to formation of thrombotic material (white clots).

Recent published reports have suggested an increased cardiovascular mortality associated with testosterone therapy. The effects of testosterone and its association with increased myocardial infarction and stroke in cisgender men under testosterone therapy for age-related hypogonadism were issued recently as an FDA Warning in accordance with recent studies [7]. It was advised to weigh the potential increased risk of major adverse cardiovascular outcomes against the potential benefits of treating hypogonadism, after FDA review of five observational studies and two meta-analyses.

On the other hand, these evidences and risks associated have also been widely disputed with contrasting evidence. Morgentaler and colleagues found no convincing evidence of increased CV risk with testosterone therapy in hypogonadism [8].

In contrast, patients on testosterone therapy for gender conversion, like the one in our case, receive significantly higher doses in comparison to that used in hypogonadism. There have been reports of increasing risk of venous thromboembolism under testosterone therapy used for gender conversion [9]. A worsening of CVD risk factors like blood pressure, insulin resistance, and lipid derangement have been reported during hormone therapy in transgender males (female to male) [10]. A direct increase in cardiovascular disease or morbidity and mortality have however not been observed [11]. The relatively young age of patients taking gender conversion therapy and short follow up durations are the limiting factors in these studies. In view of the growing transgender population, there is a need for adequately powered prospective studies with focus on the side effects of the therapy and its association with cardiovascular disease.

These factors led us to believe that testosterone therapy for gender conversion might have led to a general hypercoagulable state favoring intraarterial thrombus formation. Our patient in this case had a history of hormonal therapy with intramuscular testosterone undecanoate for gender conversion over the past 6 years. An interaction of factors like (a) vascular stasis due to episodes of paroxysmal AF, along with the (b) procoagulant tendency under high-dose testosterone therapy, and (c) endothelial injury due to the excessive smoking could also have led to the intraarterial activation of the clotting cascade leading to thrombus formation, possibly in the left atrial appendage. This is in accordance to the Virchow's triad of factors contributing to thrombus formation. The embolization of this thrombus into the coronaries could have been the cause for the acute myocardial infarction.

We believe this subpopulation of patients demand an individualized approach to further therapy. A guideline-based approach is not available here due to the very low research base. A generalized approach to therapy with the otherwise conventional CHA_2_DS_2_-VASc-Score for risk stratification in cases of paroxysmal atrial fibrillation in this subpopulation might be misleading.

Due to the high risk of recurrence of coronary embolism after the first episode, consideration was given to treating the patient with an oral anticoagulant. Despite the increased risk of recurrence, stopping of the testosterone therapy is a very delicate decision and is almost always not acceptable to the patient due to the various psychosocial factors bound with this decision.

The risk of major bleeding episodes under DOAC therapy increases with age. We believe that the risks associated with major bleeding episodes in a younger patient, like the one in this case, are tolerable compared to the risk of recurrence of the coronary embolism. Keeping all this in mind and after discussion with the patient about the potential risks, we decided to treat the patient with an oral anticoagulation—in this case with Apixaban. Regular follow-ups were advised due to the increased risk of recurrence. After 3 months and 1 year, the patient remained asymptomatic with no evidence of similar episodes under continuing testosterone therapy, as well as no major bleeding complications. However, if the patient would have reliably stopped smoking and one could manage to optimally address cardiovascular risk factors, it could have also been justifiable to omit anticoagulation or to replace it by antiplatelet therapy. But, again, evidence is low, and the decision has to be reached individually.

## References

[1] Canner J. K., Harfouch O., Kodadek L. M. (2018). Temporal trends in gender-affirming surgery among transgender patients in the United States. *JAMA Surgery*.

[2] Shibata T., Kawakami S., Noguchi T. (2015). Prevalence, clinical features, and prognosis of acute myocardial infarction attributable to coronary artery embolism. *Circulation*.

[3] Popovic B., Agrinier N., Bouchahda N. (2018). Coronary embolism among ST-segment-elevation myocardial infarction patients: mechanisms and management. *Circulation. Cardiovascular Interventions*.

[4] Ettinger P. O., Wu C. F., de la Cruz C., Weisse A. B., Ahmed S. S., Regan T. J. (1978). Arrhythmias and the "Holiday Heart": alcohol-associated cardiac rhythm disorders. *American Heart Journal*.

[5] Kirchhof P., Benussi S., Kotecha D. (2016). 2016 ESC guidelines for the management of atrial fibrillation developed in collaboration with EACTS. *European Heart Journal*.

[6] Neisius U., Northridge D. B., Cruden N. L., Denvir M. A. (2015). Myocardial infarction associated with patent foramen ovale and paradoxical embolism: a case series. *International Journal of Cardiology*.

[7] FDA Drug Safety Communication FDA cautions about using testosterone products for low testosterone due to aging; requires labeling change to inform of possible increased risk of heart attack and stroke with use. https://www.fda.gov/Drugs/DrugSafety/ucm436259.html.

[8] Morgentaler A., Miner M. M., Caliber M., Guay A. T., Khera M., Traish A. M. (2015). Testosterone therapy and cardiovascular risk: advances and controversies. *Mayo Clinic Proceedings*.

[9] Stanley K., Cooper J. (2018). Hormone therapy and venous thromboembolism in a transgender adolescent. *Journal of Pediatric Hematology/Oncology*.

[10] Streed C. G., Harfouch O., Marvel F., Blumenthal R. S., Martin S. S., Mukherjee M. (2017). Cardiovascular disease among transgender adults receiving hormone therapy: a narrative review. *Annals of Internal Medicine*.

[11] Gooren L. J. (2014). Management of female-to-male transgender persons. *Current Opinion in Endocrinology, Diabetes, and Obesity*.

